# Effects of football training on cognitive performance in children and adolescents: a meta-analytic review

**DOI:** 10.3389/fpsyg.2024.1449612

**Published:** 2024-11-12

**Authors:** Fan Mao, An Yin, Shan Zhao, Qun Fang

**Affiliations:** ^1^School of Physical Education, Qingdao University, Qingdao, China; ^2^Research Center for Youth Football, Qingdao University, Qingdao, China; ^3^Lintong Rehabilitation and Recuperation Center, Lintong, China

**Keywords:** soccer, cognition, attention, inhibitory control, working memory

## Abstract

**Background:**

The cognitive development of children and adolescents is crucial for their academic success and overall well-being. Physical activity has been linked to improved cognitive performance, but the specific effects of football training on cognitive function in this population remain unclear. This meta-analytic review aimed to comprehensively evaluate the impact of football training on cognitive performance in children and adolescents.

**Methods:**

Literature was searched through PubMed, PsycINFO, SPORTDiscus, Embase, and Web of Science. Eligible studies were randomized controlled trials (RCTs) or crossover designs assessing cognitive performance following football training interventions. Outcome measures included attention, inhibitory control, and working memory. Data synthesis and meta-analysis were performed to determine the overall effect sizes.

**Results:**

Twelve studies were included in the meta-analysis, comprising 1,574 children and 94 adolescents. Football training demonstrated moderate, statistically significant effects on attention (*Hedges’ g* = −0.77, *p* = 0.01), inhibitory control (*Hedges’ g* = −0.67, *p* = 0.02), and working memory (*Hedges’ g* = −0.44, *p* = 0.03). The findings suggest that football training positively influences cognitive performance in children and adolescents.

**Conclusion:**

Football training holds promise for enhancing cognitive function in children and adolescents, particularly in attention, inhibitory control, and working memory. Theoretical frameworks emphasizing environmental enrichment, cardiovascular fitness, and cognitive component skills help elucidate the underlying mechanisms. Future research should explore how football training compares to other sports and assess whether integrative drills that combine cognitive elements with skill practice offer greater cognitive benefits than skill training alone. These insights support the inclusion of football in educational programs to foster cognitive development.

## 1 Introduction

The cognitive development of children and adolescents holds paramount significance in the realms of education, psychology, and neuroscience (Barenberg et al., 2011; Diamond, 2015; Ferreira Vorkapic et al., 2021). Cognitive performance, encompassing key mental processes such as attention, memory, problem-solving, and decision-making (Diamond and Lee, 2011; van der Niet et al., 2014), plays a pivotal role in academic achievement, social interaction, and overall well-being during these formative years (Diamond and Lee, 2011; Donnelly et al., 2016). This period of developmental plasticity provides a unique opportunity for cognitive growth, as the brain’s structure and function can be positively influenced by enriching environments, such as physical activity (Kobilo et al., 2011). The plasticity inherent in cognitive development during childhood and adolescence underscores its profound implications for future mental health, academic success, and social interactions (Ahmed et al., 2021; Blakemore and Choudhury, 2006).

The relationship between physical activity and cognitive performance has garnered substantial attention, with numerous studies and systematic reviews attesting to the positive impact of physical activity interventions on various cognitive domains (Bidzan-Bluma and Lipowska, 2018; de Greeff et al., 2018; Donnelly et al., 2016; Singh et al., 2019). The growing interest in understanding the connection between physical activity and cognitive outcomes has extended to exploring the effects of football training on cognitive performance in children and adolescents (Magistro et al., 2022; Williams et al., 2020). Football, distinguished by its multidimensional cognitive demands that encompass decision-making, perception, observation, and action, provides a unique platform to investigate potential cognitive benefits arising from sports participation (Hicheur et al., 2017). The cognitively challenging nature makes soccer more than a sport for fun or health promotion. Cognitive capabilities are highly plastic during childhood and adolescence (Gogtay et al., 2004). Soccer play provides children and adolescents with affordances to stimulate cognitive development. Therefore, it is crucial to conduct an in-depth investigation into the specific effects of football training on cognitive performance, including aspects such as executive function, attention, and inhibition.

Cross-sectional studies have provided compelling evidence of the cognitive advantages associated with youth soccer participation, revealing that young athletes often outperform their non-athlete peers in various cognitive domains. For instance, a study by Moratal et al. (2020) found that adolescent soccer players displayed superior performance in tasks assessing executive control, attention, and alerting. These players exhibited heightened cognitive flexibility, allowing them to adapt their thinking and strategies in response to rapidly changing game situations. Furthermore, Yongtawee et al. (2022) demonstrated that youth soccer players outperformed athletes from non-cognitive demanding sports, such as shooting, particularly in tests of Flanker Test and Trail Making Test, highlighting the unique cognitive demands of soccer. In addition to these findings, Ballester et al. (2015) reported that soccer players showed enhanced vigilance, indicating their ability to maintain focused attention over prolonged periods, which is crucial for both academic and athletic success. Contrastingly, experimental studies have provided more definitive insights into the causal relationships between football training and cognitive enhancement. A notable randomized control trial conducted by Lind et al. (2018) involved an 11-month intervention where children aged 11–12 participated in structured soccer training sessions. This study revealed that the soccer group exhibited significant improvements in cognitive performance such as attention and working when compared to normal control group. The results indicated not only enhanced cognitive flexibility but also better impulse control, essential for both academic settings and real-life decision-making scenarios. Similarly, Alesi et al. (2016) found that participants in a soccer training program showed substantial gains in executive function and attention after 6 months of intervention. These studies underscore the importance of structured physical activity in fostering cognitive development, particularly in dynamic environments like soccer, which require quick thinking and strategic planning. However, there remains a lack of comprehensive integration regarding the impact of football training on cognitive performance in children and adolescents. This meta-analysis aims to provide a thorough synthesis of the existing evidence to address this issue.

One of the key strengths of this meta-analytic review is the integration of both qualitative and quantitative methodologies for analyzing the data. Meta-analysis serves as a robust tool for synthesizing findings across diverse studies, enabling a clearer understanding of the overall effects of interventions through the calculation of effect sizes and the exploration of potential moderators that might influence these effects (Borenstein et al., 2011). In this review, we systematically assess the quantitative data derived from studies investigating the impact of football training on cognitive outcomes such as attention, inhibitory control, and working memory. In contrast, qualitative analysis explores the contextual features of the specific cognitive domains targeted (Croker, 2009). This dual methodology ensures a holistic understanding of how football training influences cognitive development. This study not only contributes to our understanding of the cognitive effects of football training but also offers practical implications for educators, coaches, and policymakers seeking to incorporate physical activity into cognitive development programs.

This meta-analytic review seeks to fill a significant gap in the literature by providing a comprehensive evaluation of the effects of football training on cognitive performance in children and adolescents. By incorporating both qualitative and quantitative analyses, we aim to provide a robust synthesis of the evidence, highlighting both the potential benefits and the limitations of current research. This study not only contributes to our understanding of the cognitive effects of football training but also offers practical implications for educators, coaches, and policymakers seeking to incorporate physical activity into cognitive development programs. With the increasing focus on cognitive health in youth, understanding how structured physical activity interventions like football training can contribute to cognitive performance is of critical importance for both research and practice.

## 2 Methods

This review was conducted in accordance with the guidelines from Preferred Reporting Items for Systematic Reviews and Meta-Analysis (PRISMA) (Moher et al., 2009) and Cochrane Collaboration handbook (Cumpston et al., 2019). The protocol of this meta-analytic was registered in the International Prospective Register of Systematic Reviews (PROSPERO; CRD42024592698).

### 2.1 Search strategy

The electronic databases PubMed, PsycINFO, SPORTDiscus, Embase, and Web of Science were searched for original research published in peer-reviewed journals by January 2024. According to the main purpose of the current review, which aims to examine influence of football training on cognitive performance of children and adolescents, the keywords for literature search involve three categories including sports, cognition, and population. Snowball search was used to track down any study that might not be identified in the initial search. Specifically, the search equation was structured as follows: ((“Football” OR “Soccer” OR “Football Training” OR “Soccer Training” OR “Football Practice” OR “Soccer Practice”) AND (“Cognitive Function*” OR “Cognition” OR “Executive Function*” OR “Cognitive Performance” OR “Attention” OR “Memory” OR “Working Memory” OR “Inhibitory Control” OR “Cognitive Processing” OR “Cognitive Flexibility” OR “Cognitive Development”) AND (“Child*” OR “Adolescent*” OR “Youth” OR “Teen*” OR “Young People” OR “Pediatric*” OR “School-Age Children”) AND (“Randomized Controlled Trial” OR “RCT” OR “Intervention” OR “Experimental Study” OR “Clinical Trial” OR “Controlled Trial”)).

Outcomes of the initial search were screened by an overall examination on the title to remove duplicates and irrelevant articles. The second phase of screening was conducted by analysis on the abstracts. For the articles that passed the first two phases, a full-text evaluation was performed to determine the included studies of the current review. Two authors (FM and QF) independently worked on the literature screening and selection. Any disagreement on the eligibility of an article was resolved by discussing with other authors to reach a consensus.

### 2.2 Eligibility criteria

Studies considered eligible for inclusion should be published in peer-reviewed journals. The language was restricted to English. Further requirements on the eligible criteria followed the guide of Population, Intervention, Comparator, Outcomes, and Study (PICOS). Specific eligibility criteria are shown in Table 1. First, participants of the included studies should be healthy children and adolescents. Second, intervention was designed upon football practice. Third, comparisons were made between groups at different time points. Fourth, outcomes measured cognitive performance such as attention, working memory, inhibitory control, etc. Fifth, the included studies should adopt an experimental design which indicated performance change associated with football practice.

**Table 1:** Eligibility criteria.

	Inclusion	Exclusion
Population	The primary population of interest in this meta-analysis is healthy children and adolescents aged 6–18 years.	Studies focusing on individuals with neurodevelopmental or cognitive disorders were excluded.
Intervention	The interventions of interest include both acute and long-term football-related activities. Eligible interventions consist of structured training sessions, football matches, or basic football skill exercises.	Other physical activities or sports, as well as studies without a primary focus on football, were excluded.
Comparison	Studies were included if they featured a comparison group. This comparison could involve no intervention, wait-list controls, or alternative forms of physical activity such as running, swimming, or other team sports.	Absence of a control group.
Outcomes	The primary outcomes of interest were cognitive performance measures, including executive function (inhibitory control, cognitive flexibility, working memory) and attention.	Studies that did not report specific cognitive outcomes were excluded, as cognitive performance is the focus of this meta-analysis.
Study design	We included randomized controlled trials (RCTs), crossover studies, and controlled trials with baseline and post-intervention measurements.	Cross-sectional studies, case reports, and studies without a control group were excluded.

On the other hand, studies were excluded for any of the following reasons: (1) review, book chapter, commentaries, or proceedings; (2) articles in which data could not be obtained or extracted for estimating effect size even after contacting the authors; (3) studies that did not assess cognitive functions associated with football practice; (4) intervention protocols that combined football with other sports; and (5) non-English articles.

### 2.3 Data extraction and synthesis

Data extraction was performed independently by two reviewers (FM and QF) using a standardized data extraction form. Basic information including study design, sample size, and age of the participants provided an overall examination of the reviewed studies. In addition, intervention protocols presented details of football-based sessions and control sessions, with particular attention to the time per session, frequency, and duration of the programs. Further analysis focused on outcome measures of cognitive functions in individual studies. Based on the empirical data of cognitive performance, meta-analysis was conducted to provide evidence regarding the effects of football play on cognitive functions.

The quantitative data were synthesized according to the categories of cognitive functions. Effect sizes which assessed the same cognitive domain were combined to compute an overall effect. Multiple effects in the same study were addressed by the following steps. First, if multiple results were reported by one cognitive assessment, the result of the more cognitive demanding condition was extracted (Álvarez-Bueno et al., 2017; Xue et al., 2019). Second, for the tests which reported both accuracy and time, accuracy was selected as a representation of cognitive performance. Therefore, in the study which applied Flanker task to assess inhibitory control, accuracy of incongruent trials would be extracted in data synthesis. Any disagreements were discussed with a third author (AY) until a consensus was achieved.

### 2.4 Statistical analysis

Statistical analysis was conducted in Comprehensive Meta-Analysis 3.3 (BioStat Inc., Englewood, NJ, United States). As a measure of effect size to explore differences between exergaming groups and control groups, Hedges’ g was calculated based on sample size, mean, and standard deviation of both experimental group and control group at pre-test and post-test. The magnitude of Hedges’ g was interpreted using Cohen’s guidelines, distinguishing between small (<0.2), moderate (0.5), and large (>0.8) effect sizes (Cohen, 1988).

A random effect model would be used in the case of a high heterogeneity. The heterogeneity was assessed by *I*^2^ and *p*-value for *Q* statistic, with the *I*^2^ values of 25, 50, and 75% indicating small, moderate, and large heterogeneity, respectively (Xue et al., 2019). Egger’s regression test was performed to assess publication bias in the reviewed literature. A two-tailed test with *p*-value less than 0.05 was considered significant publication bias. Finally, sensitivity analyses were conducted by excluding one study at a time from the meta-analysis.

### 2.5 Risk of bias assessment

To ensure a comprehensive evaluation of the methodological quality of the included studies, the Risk of Bias 2 (RoB 2) tool was employed for assessing RCTs, while the ROBINS-I tool was utilized for non-randomized interventions (Higgins et al., 2020). The RoB 2 tool provides a robust framework for evaluating risks associated with random sequence generation, allocation concealment, blinding, and other potential biases, ensuring clarity in the assessment process (Sterne et al., 2019). Two independent researchers conducted the risk assessments, and inter-rater agreement was quantified using Cohen’s kappa to evaluate the consistency of their evaluations. Any discrepancies were discussed and resolved collaboratively, with the final results reported in a format that visualizes the risk of bias across studies.

## 3 Results

**Figure 1:**
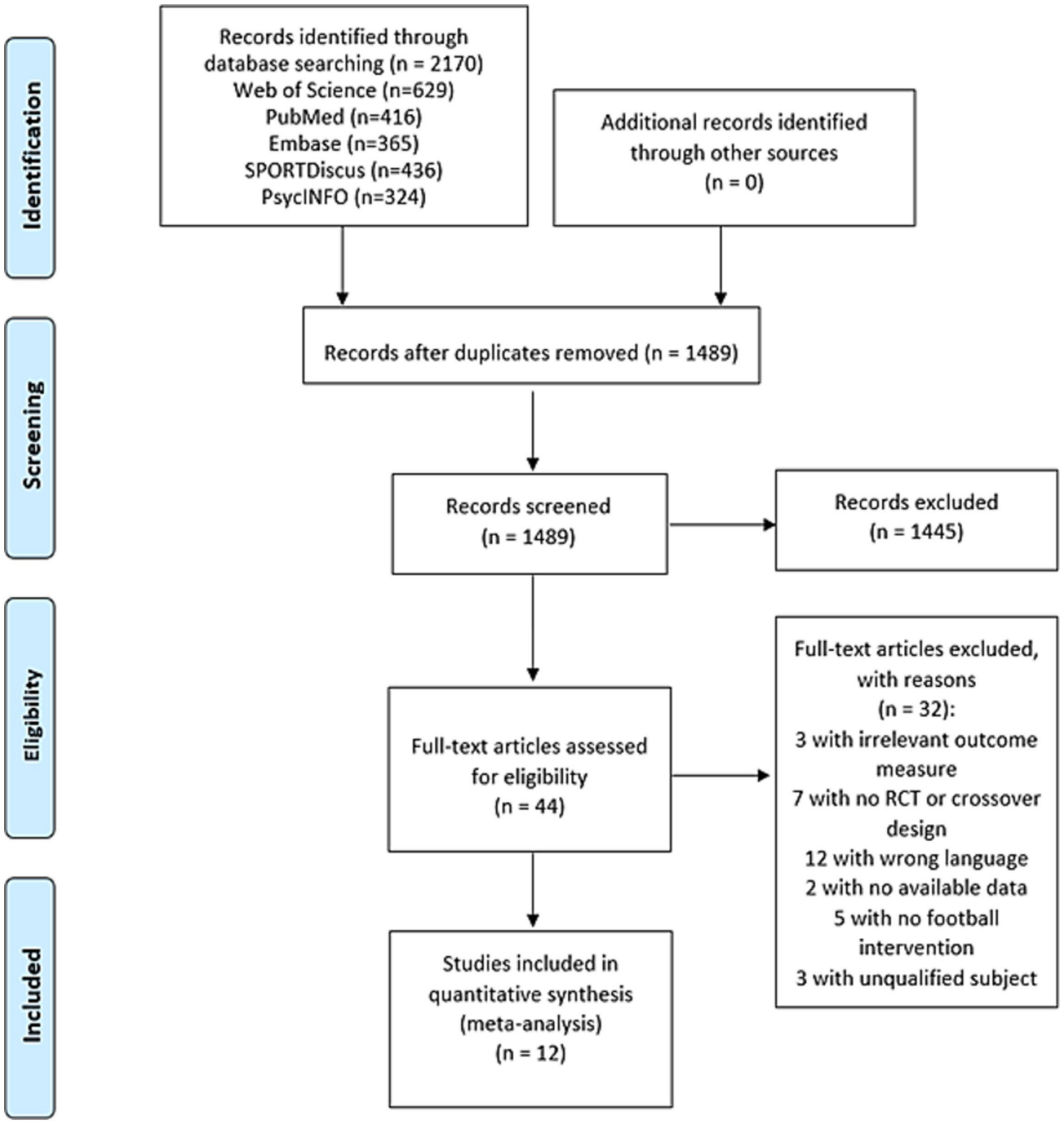
Flowchart of literature search and study selection.

The initial search retrieved 2,170 peer-reviewed articles. After removing duplications and reviewing the titles, 1,489 articles were eligible for further screening. Through a careful reading of the abstracts, 44 articles were eligible for thorough examination. Finally, 12 studies were included in the meta-analysis, comprising 8 randomized controlled trials (RCTs) and 4 crossover studies. Among the initial pool of 44 articles, 32 were excluded due to the absence of relevant cognitive outcome measures, non-RCT or crossover design, non-English language, missing data, ineligible participants, or lack of football intervention. Figure 1 displays the flow of study selection.

### 3.1 Characteristics of included studies

Overall, 12 studies were included in the meta-analysis, and the characteristics of these studies are summarized in Table 2. Among these studies, eight were RCTs, and four were crossover designs. The total sample size included 1,574 children and 94 adolescents aged from 8 to 18 years, with 1,319 participants assigned to the football intervention group and 511 participants assigned to the control group.

**Table 2:** Characteristics of the included studies.

Study	Design	Sample size	Age (SD)	Outcome measures	Intervention protocol
Alesi et al. (2015)	RCT	*N* = 44 EG: 24, CG: 20	EG: 8.78 (1.13) CG: 9.41 (0.96)	Attention: visual Discrimination test	EG: football exercise 75 min/session, 2 times/wk., 24 weeks CG: sedentary
Alesi et al. (2016)	RCT	*N* = 46 EG: 24, CG: 22	EG: 8.78 (1.12) CG: 9.25 (0.85)	Attention: visual discrimination test; Inhibitory control: tower of London task; Working memory: forward and backward digit span tests	EG: football exercise 75 min/session, 2 times/wk., 24 weeks CG: sedentary
Chen et al. (2021)	RCT	*N* = 52 EG: 26, CG: 26	EG: 11.35 (0.19) CG: 11.25 (0.32)	Attention: Attention scale for elementary school children test	EG: football exercise 40 min/session, 5 times/wk., 8 weeks CG: sedentary
Gilbert et al. (2023)	Crossover	*N* = 76 EG: 76, CG: 76	12.2 (0.4)	Attention: visual search; Inhibitory control: Stroop test; Working memory: Sternberg paradigm	EG: 60 min football lesson CG: 60 min academic lesson
Kolovelonis et al. (2022)	Crossover	*N* = 99 EG: 61, CG: 38	9.37 (0.59)	Inhibitory control: Stroop test and Flanker test	EG: football games 45 min/session, 2 times/wk., 4 weeks CG: normal PE sessions
Kolovelonis and Goudas (2023)	Crossover	*N* = 67 EG: 34, CG: 33	9.94 (0.60)	Inhibitory control: design fluency test	EG: 45 min football lesson CG: waiting-list
Lind et al. (2018)	RCT	*N* = 931 EG:838, CG: 93	EG: 11.9 (0.4) CG: 11.8 (0.2)	Attention: detection and identification task; Working memory: 1-back paradigm	EG: football exercise 45 min/session, 2 times/wk., 44 weeks. CG: normal physical activity
Lind et al. (2019)	RCT	*N* = 54 EG: 27, CG: 27	EG: 11.7 CG: 11.9	Inhibitory control: Flanker test	EG: 20 min high-intensity small-sided real football games CG: watch the recording
Magistro et al. (2022)	RCT	*N* = 100 EG: 50, CG: 50	8–9 years M (SD): NR	Attention: selective visual attention test; Working memory: Rey word recognition test	EG: 60 min football session CG: resting
Shi et al. (2021)	RCT	*N* = 94 EG: 41, CG: 53	EG: 18.26 (0.52) CG: 18.49 (0.75)	Working memory: 2-back task	EG: football learning 30 min/session, 7 times/wk., 40 weeks CG: normal activity
Wen et al. (2021)	RCT	*N* = 69 EG: 32, CG: 37	EG: 10.9 (0.3) CG: 10.9 (0.4)	Inhibitory control: go/no go task; Working memory: 2-back task	EG: 40 min football training. CG: 40 min watch cartoons
Williams et al. (2020)	Crossover	*N* = 36 EG: 36, CG: 36	12.6 (0.5)	Inhibitory control: Stroop test; Working memory: Sternberg paradigm	EG: 60 min football session CG: 60 min rest

N, number of participants; SD, standard deviation; NR, not reported; RCT, randomized controlled trial; EG, experimental group; CG, control group.

The included studies mainly examined three categories of cognitive functions including inhibitory control (*N* = 7), working memory (*N* = 7), and attention (*N* = 6). The exercise intervention protocols varied across the studies, with 6 studies employing single acute interventions of 20–60 min, and another 6 studies implementing chronic interventions lasting over 4 weeks. Meta-analyses were implemented to determine the effects of football training on the cognitive functions in children and adolescents.

### 3.2 Effects of football intervention on attention

Six studies assessed the effects of football training on attention, with 1,038 participants receiving football intervention and 287 participants assigned to the control group. The most commonly used paradigm (*N* = 5) required participants to initiate a quick response following presentation of visual stimuli (Alesi et al., 2016; Alesi et al., 2015; Gilbert et al., 2023; Lind et al., 2018; Magistro et al., 2022). One study assessed attention by means of a 10-item scale (Attention Scale for Elementary School Children) on focused attention, sustained attention, selective attention, alternating attention, and divided attention (Chen et al., 2021). The findings were consistent given that all the studies reported benefits in attentional performance of the football intervention programs compared with the control session. A meta-analysis was conducted to provide empirical evidence regarding the effects of football play on attention.

**Figure 2:**
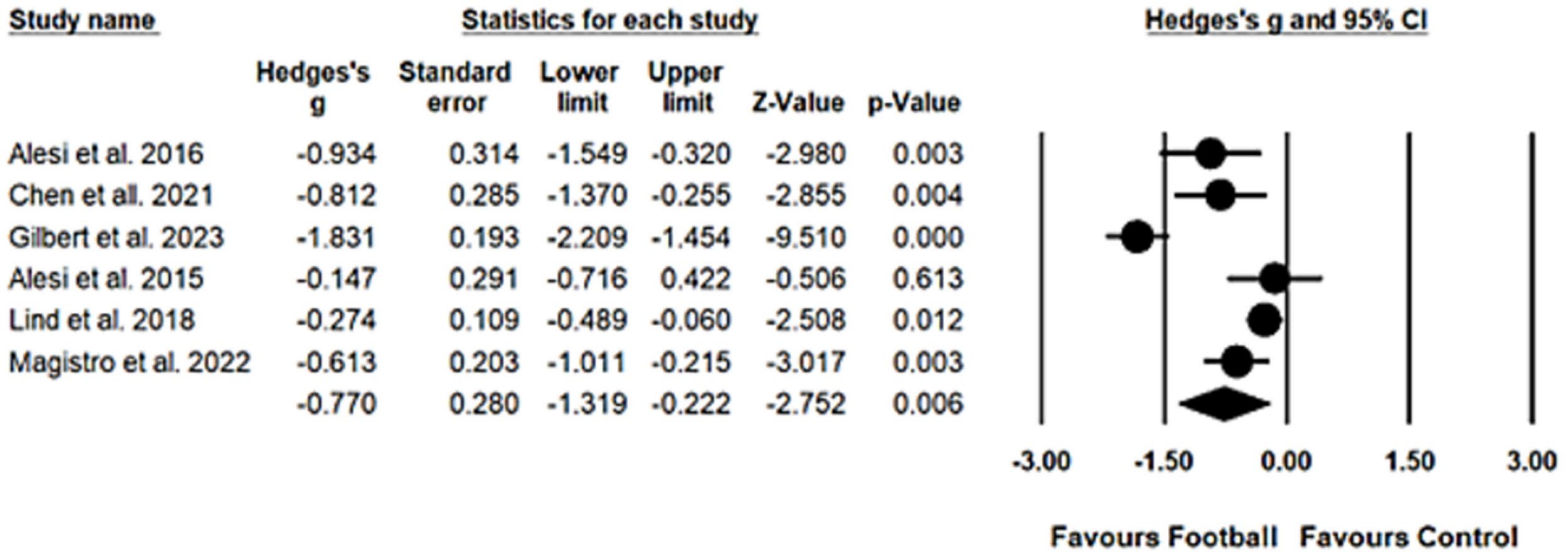
Forest plot for attention.

Due to a substantial heterogeneity (*Q_5_* = 53.60, *I^2^* = 90.67, *p* < 0.001), a random effect model was applied to calculate the overall effect. A moderate, statistically significant effect was identified (*Hedges’ g* = −0.77, 95% *CI* = −1.32 to-0.22, *p* = 0.01), which substantiated the beneficial effects of football play on attention. Egger’s regression test suggests a low risk of publication bias (*t_4_* = 0.79, *p* = 0.48). The result of meta-analysis on attention was displayed in Figure 2.

### 3.3 Effects of football intervention on inhibitory control

A total of 7 studies involving 447 participants (football = 290, control = 269) examined the impact of football intervention on inhibitory control. The majority of paradigms (*N* = 5) required participants to respond rapidly and accurately under conditions of stimulus interference (Gilbert et al., 2023; Kolovelonis et al., 2022; Lind et al., 2019; Wen et al., 2021; Williams et al., 2020). One study assessed inhibitory control using the Design Fluency Test, in which participants were asked to generate as many unique designs as possible within a limited time period (Kolovelonis and Goudas, 2023). Another study utilized the Tower of London task, wherein participants were required to coordinate the positions of balls and goals (Alesi et al., 2016). The most of study findings reported beneficial effects of football intervention programs on inhibitory control performance. However, two studies indicated that football interventions did not yield positive effects on inhibitory control compared to the control group, attributed to excessive exercise intensity leading to reduced prefrontal cortical neural activity in the brain (Lind et al., 2019; Williams et al., 2020).

**Figure 3:**
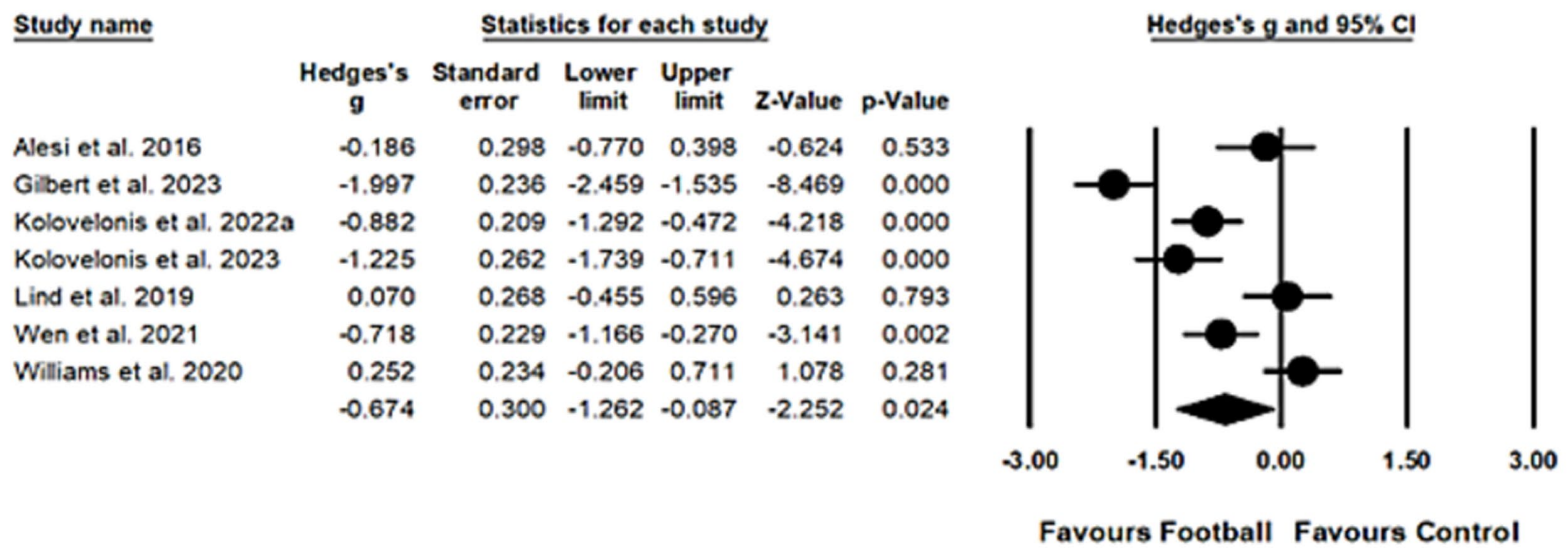
Forest plot for inhibitory control.

Due to substantial heterogeneity (*Q_6_* = 63.79, *I^2^* = 90.44, *p* < 0.001), a random-effects model was employed to calculate the overall effect. A moderate and statistically significant effect was identified (*Hedges’ g* = −0.67, 95% *CI* = −1.26 to-0.09, *p* = 0.02), confirming the beneficial effects of football play on inhibitory control. Egger’s regression test suggests a low risk of publication bias (*t_5_* = 0.62, *p* = 0.56). The result of meta-analysis on inhibitory control was presented in Figure 3.

### 3.4 Effects of football intervention on working memory

In the 7 studies that examined the effects of football intervention on working memory, a total of 1,448 participants were included, with 1,097 in the football training group and 367 in the control group. Three studies utilized the n-back paradigm to assess working memory, requiring participants to rapidly and accurately respond to previous stimuli (Lind et al., 2018; Shi et al., 2021; Wen et al., 2021). In two studies, participants were required to rapidly identify target items using the Sternberg paradigm (Gilbert et al., 2023; Williams et al., 2020). The remaining two studies mandated participants to accurately recall target words and numbers (Alesi et al., 2016; Magistro et al., 2022). The most of study outcomes substantiate the beneficial effects of football interventions on working memory performance. Only one study reported no positive impact of football intervention on working memory compared to the control group, possibly due to excessive football exercise intensity resulting in diminished neural activity (Williams et al., 2020).

**Figure 4:**
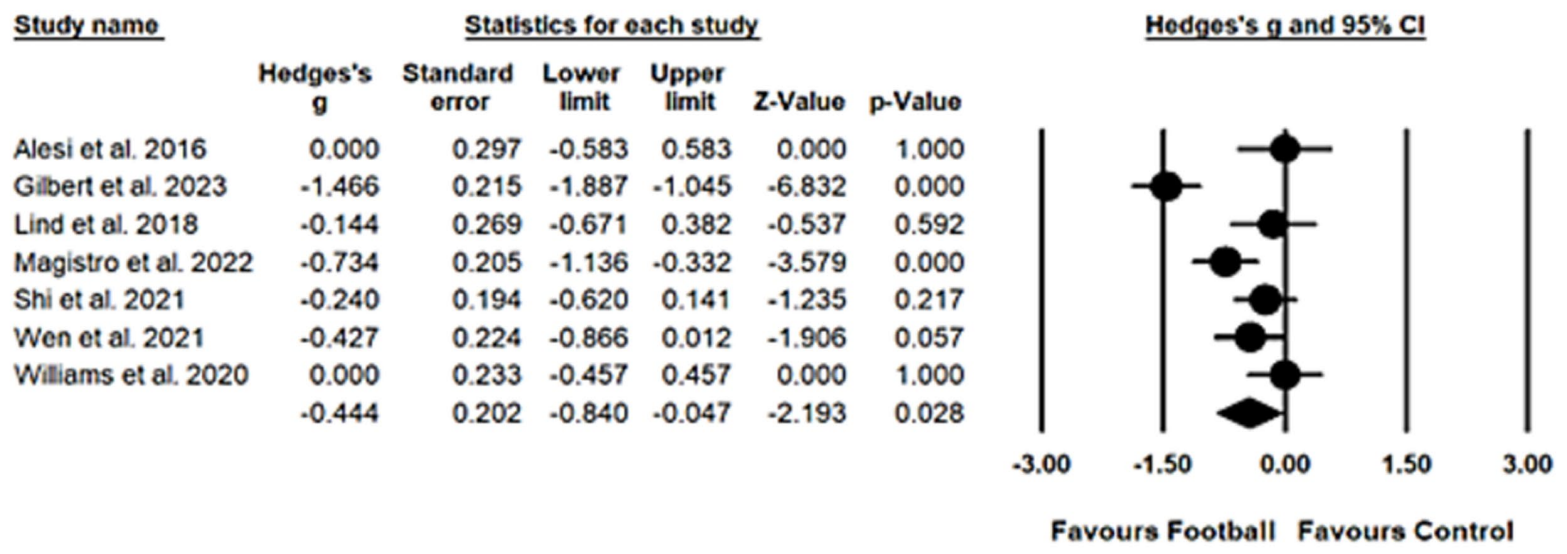
Forest plot for working memory.

A random-effects model was employed to compute the overall effect size, given substantial heterogeneity (*Q_6_* = 32.63, *I^2^* = 81.61, *p* < 0.001). A moderate and statistically significant effect was identified (*Hedges’ g* = −0.44, 95% *CI* = −0.84 to −0.05, *p* = 0.03), providing evidence for the beneficial effects of football training on working memory. Egger’s regression test suggests a low risk of publication bias (*t_5_* = 0.98, *p* = 0.37). The result of meta-analysis on working memory was shown in Figure 4.

### 3.5 Sensitivity analysis

One study Gilbert et al. (2023) was major contributors to the high heterogeneity by the sensitivity analysis in attention, inhibitory control, and working memory. After excluding the study, the pooled *Hedges’ g* of attention was −0.504 (95% CI = −0.782 to −0.227), and heterogeneity was medium (*I*^2^ = 50.9%, *p* < 0.001); the pooled *Hedges’ g* of inhibitory control was-0.452 (95% CI = −0.921 to −0.012), and heterogeneity was large (*I*^2^ = 82.0%, *p* = 0.056); the pooled *Hedges’ g* of working memory was −0.287 (95% CI = −0.521 to −0.053), and heterogeneity was medium (*I*^2^ = 36.8%, *p* = 0.016). The sensitivity analysis is shown in Table 3.

**Table 3:** Sensitivity analysis.

Study	ES	[95% Conf. interval]	*I*^2^ (%)	*P*
A. Attention				
Alesi et al. (2016)	−0.740	−1.367, −0.112	92.4	0.021
Chen et al. (2021)	−0.762	−1.401, −0.122	92.5	0.020
Gilbert et al. (2023)	−0.504	−0.782, −0.227	50.9	<0.001
Alesi et al. (2015)	−0.887	−1.508, −0.265	92.1	0.005
Lind et al. (2018)	−0.882	−1.491, −0.272	87.2	0.005
Magistro et al. (2022)	−0.802	−1.491, −0.113	92.5	0.023
B. Inhibitory control				
Alesi et al. (2016)	−0.752	−1.408, −0.096	91.6	0.025
Gilbert et al. (2023)	−0.452	−0.921, 0.012	82.0	0.056
Kolovelonis et al. (2022)	0.638	−1.348, 0.073	91.9	0.079
Kolovelonis and Goudas (2023)	0.583	−1.246, 0.080	91.4	0.085
Lind et al. (2019)	−0.796	−1.426, −0.166	90.6	0.013
Wen et al. (2021)	−0.666	−1.369, 0.037	92.0	0.063
Williams et al. (2020)	−0.833	−1.410, −0.255	88.3	0.005
C. Working memory				
Alesi et al. (2016)	−0.509	−0.939, −0.079	83.1	0.021
Gilbert et al. (2023)	−0.287	−0.521, −0.053	36.8	0.016
Lind et al. (2018)	−0.489	−0.153, 0.945	83.7	0.031
Magistro et al. (2022)	−0.391	−0.856, 0.075	83.8	0.100
Shi et al. (2021)	−0.477	−0.946, −0.009	83.6	0.046
Wen et al. (2021)	−0.444	−0.914, 0.026	84.6	0.064
Williams et al. (2020)	−0.518	−0.948, −0.087	81.8	0.018

### 3.6 Methodological quality assessment

**Figure 5:**
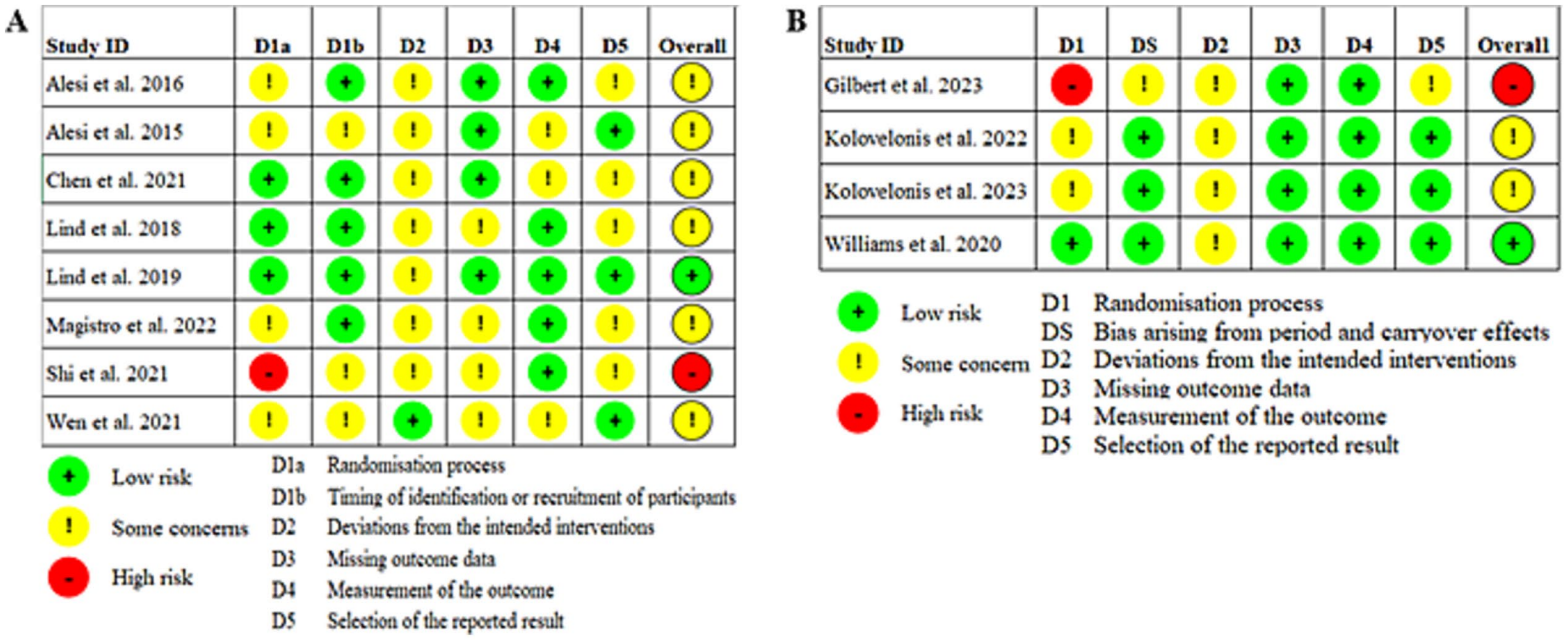
Risk-of-bias assessment. (A) Risk of bias in RCT. (B) Risk of bias in crossover.

Figure 5 presents the risk of bias plot for the included studies. The risk of bias assessment was conducted by two reviewers, resulting in a Cohen’s kappa value of 0.80, which indicates a substantial level of inter-rater agreement and reliable consistency in their evaluations. In the risk of bias assessment for randomized controlled trials, more than half of the studies exhibited some degree of risk during the randomization process, which may have introduced bias in the post-intervention outcomes. Additionally, most studies showed potential issues with deviations from the intended interventions. However, in terms of outcome measurement, the majority of studies were categorized as low risk. Overall, the risk of bias was relatively high. In the assessment of risk of bias in crossover trials, most studies demonstrated low risk in outcome data, outcome measurement, and selective reporting. Nevertheless, the randomization process posed a high risk. In general, the results of the crossover trials indicated a high overall risk.

## 4 Discussion

The current study investigates the influences of football training on cognitive performance in children and adolescents. Meta-analyses provide empirical evidence for football training on cognitive performance, implying neurophysiological changes underlying the effects. The study demonstrates a moderate-sized beneficial impact of football training on cognitive performance. Specifically, attention, inhibition control, and working memory showed substantial changes after engaging in football training. These findings underscore the significance of football training in promoting cognitive development in children and adolescents, offering insights into the neural mechanisms and factors contributing to the observed effects.

### 4.1 Neural mechanisms for cognitive promotion

The cognitive improvement in attention, inhibitory control, and working memory following football training may be attributed to various neurochemical and neurophysiological mechanisms. Physical activity, including football, induces neuroplastic changes that positively influence cognitive functions. Increased levels of brain-derived neurotrophic factor (BDNF), a key neurotrophin associated with synaptic plasticity and cognitive function, have been linked to exercise-induced cognitive improvements (Voss et al., 2013). Furthermore, exercise promotes neurotransmitter release, including dopamine and serotonin, which play vital roles in attention and inhibitory control (Roig et al., 2013). Another factor that contributes to the improved cognitive performance is prefrontal cortex (PFC), a critical brain region for executive functions, experiences notable developmental changes during adolescence (Gogtay et al., 2004). Football play demands strategic thinking, planning, and decision-making which stimulate and refine the neural circuits in the PFC. The observed improvements in inhibitory control could be attributed to enhanced prefrontal cortical activity, supporting the suppression of irrelevant information and impulsive responses (Best, 2010).

The cardiovascular and oxygenation effects of football training also play a crucial role in cognitive improvements. Increased blood flow to the brain during physical activity enhances oxygen and nutrient delivery, supporting optimal brain function (Hillman et al., 2008). Aerobic exercise, inherent in football, has been associated with improved cardiovascular fitness, leading to enhanced cerebrovascular health and cognitive performance (Chaddock et al., 2010). Additionally, the release of vascular endothelial growth factor (VEGF) during exercise contributes to neurovascular coupling, promoting angiogenesis and neurogenesis, further supporting cognitive functions (Fabel et al., 2003). Collectively, these neurochemical changes highlight the intricate relationship between physical activity, neurobiology, and cognitive function (Dishman et al., 2006; McMorris and Hale, 2012).

### 4.2 Theoretical understandings of cognitive benefits associated with football

The varied and intricate environmental stimuli provided by football training align with the environmental enrichment hypothesis (Nithianantharajah and Hannan, 2006; Sale et al., 2009). This hypothesis posits that exposure to complex and stimulating environments fosters neural growth and connectivity (Rosenzweig and Bennett, 1996). The dynamic and unpredictable nature of football, combined with the need for constant adaptation to changing game scenarios, creates an enriched environment for cognitive stimulation. Enhanced attention and working memory could be linked to the neural changes facilitated by the enriched environment of football training (Fabel and Kempermann, 2008). The continuously changing and unpredictable sports environment provides an ideal setting for the cognitive development of children and adolescents (Kolb and Gibb, 2011). Responses and adaptive actions in this dynamic environment contribute to the improvement of motor cognition and sports experience, subsequently fostering improvements in general cognitive explicit behaviors through cognitive transfer (Kolb and Gibb, 2011). Increasing evidence suggests that engaging in open-skills and strategic sports confers more cognitive advantages in cognitive performance tasks compared to closed-skills sports (Gallotta et al., 2015; Krenn et al., 2018; Shi and Feng, 2022; Wang et al., 2016). A study comparing the cognitive performance effects of football and track and field sports found that football players outperformed track and field athletes in attention and executive control (Rahimi et al., 2022). Verburgh et al. (2016) and Moratal et al. (2020) discovered that children aged 8–12 who regularly participated in football training exhibited more significant advantages in working memory, attention, and information processing speed.

When we exploring potential causal relationships between physical activity and cognitive processes, the cardiovascular fitness hypothesis posits that cardiovascular health serves as the physiological mediator explaining the cognitive benefits of physical activity (North et al., 1990). Engaging in regular football training may lead to improved aerobic fitness in adolescents (Ballester et al., 2018). Studies have shown that football practice can enhance musculoskeletal, metabolic, and cardiovascular adaptations in adults (Krustrup et al., 2010). In this regard, the cardiovascular fitness hypothesis assumes that higher cardiovascular capacity, inherent to regular practice of physical exercise, accounts for cognitive improvements in individuals who exercise regularly due to physiological adaptations (Voss et al., 2011). Some instances in this context include increased VO_2_ max, elevated brain-derived neurotrophic factor (BDNF), and enhanced cerebral blood flow (North et al., 1990). Specifically, Hillman et al. (2009) and Pontifex et al. (2011) utilized various types of flanker tasks to investigate this relationship. They observed that preadolescent children with lower levels of aerobic fitness displayed lower accuracy compared to those with higher fitness levels.

Cognitive improvement following football training interventions can be explained through the cognitive component skills theory (Mann et al., 2007; Voss et al., 2010). This theory emphasizes that athletes exhibit enhanced cognitive performance in cognitive measurements outside the sporting environment. According to Voss et al. (2010), this hypothesis posits that “sport training is a medium of experience-dependent brain plasticity or cognitive training resulting in more efficient brain networks,” leading to enhanced perceptual-cognitive skills. It is crucial to note that optimal performance in externally-paced sports like football not only requires good physical fitness but also the ability to rapidly adapt and respond to complex and ever-changing situations. Therefore, systematic and structured practice of externally-paced sports, such as football, involves learning and practicing fundamental cognitive skills to manage these situations. The cognitive component skills theory implies that such learning will transfer to other general or specific domains, as demonstrated in previous research (Ballester et al., 2019; Best, 2010; Williams et al., 2011). In this regard, Moratal et al. (2020) observed that children undergoing systematic football training exhibited faster reactions and better executive control compared to the control group. The specific demands of the football environment may necessitate this “cognitive specialization” in cognitive performance, as players are exposed to situations where they must select relevant stimuli in complex environments and make decisions among several possible options under high time pressure (Voss et al., 2010; Williams et al., 1999). Another study demonstrated that football players exhibited more flexible visual attention during exercise (Pesce et al., 2007). The stimulating open environment of sports training enhances cognitive skills, which may influence joint cognitive functions in turn. This series of studies suggests that the cognitive demands inherent in sensorimotor learning (Myer et al., 2015) and performing complex motor tasks (Chang et al., 2017) are crucial factors contributing to the positive correlation between physical activity and cognition.

### 4.3 Implications for educational practices and interventions

The cognitive benefits associated with football play have promising implications for educational practice. The reasons for children and adolescents engaging in football are not limited to the benefits in physical fitness and health, but also cognitive promotion. Therefore, the first implication based on the primary findings is a balanced development for both motor skills and cognitive performance. Empirical evidence has shown effectiveness in enhancing attentional control, inhibitory functions, and working memory. The cognitive component skills theory supports the idea that the cognitive processes engaged in football closely align with those required in specific cognitive tasks, potentially facilitating the transfer of cognitive benefits to academic and cognitive domains. Educational institutions may consider incorporating structured football training sessions into physical education curricula.

Additionally, the positive impact of football on inhibitory control has implications for interventions targeting executive functions, which are integral to academic achievement and life success (Diamond, 2013). Schools can explore the integration of cognitive training programs inspired by the cognitive demands of football. An innovative opinion recommends integrating cognitive elements into basic drill practice (Mao et al., 2024). Training sessions should consist of tasks that require rapid decision-making, response inhibition, and strategic planning, mirroring the cognitive processes engaged in football. For instance, training programs focusing on decision-making under time constraints, spatial awareness, and adapting to unpredictable situations mirror the cognitive demands of football. By tailoring interventions to engage these specific cognitive processes, educators can potentially optimize the cognitive benefits derived from football training. The principle of specificity suggests that activities closely aligned with cognitive goals are more likely to yield optimal outcomes (Scharfen and Memmert, 2021). Therefore, integrating football into educational programs could offer a multifaceted approach to cognitive development. The specific cognitive demands of football, such as decision-making, strategic thinking, and motor coordination, position it as an ideal candidate for targeted interventions.

### 4.4 Limitations and future directions

However, limitations of the existing studies must be acknowledged. The substantial heterogeneity observed among the included studies indicates variability in intervention protocols, durations, and participant characteristics, which may influence the overall effect sizes. Although publication bias was assessed and found to be low, the possibility of unreported negative results cannot be entirely excluded. Furthermore, the lack of detailed reporting on blinding procedures and allocation concealment in many studies introduces a potential risk of bias. Future research should aim to standardize intervention protocols to better understand the impact of football training on cognitive performance in children and adolescents.

Another direction for subsequent research work is to test the assumption whether physical activity associated with a high level of cognitive engagement promotes cognitive performance more than that with limited cognitive demand. Efforts to substantiate the assumption may focus on two possible approaches. First, the multi-cognitive nature of football makes it an appropriate model to be compared with other sports regarding the effects of participation on cognitive promotion. Existing studies have made such comparisons between football players and athletes in track-and-field (Rahimi et al., 2022), boxing, and shooting (Yongtawee et al., 2022). Evidence from the cross-sectional studies suggests favorable outcomes of football play to other sports in cognitive promotion. In addition to the investigations across sports, integrative drills that account for both cognitive elements and skills can be compared with the skill practice alone. A series of football drills can be designed in consistence with the idea of integrative training. More importantly, comparative research in future helps researchers to understand whether the basic drills integrating cognitive elements can be more effective than basic skill training alone (Casella et al., 2022).

## 5 Conclusion

In conclusion, the meta-analysis provided robust evidence to substantiate football play for cognitive promotion in as attention, inhibitory control, and working memory. Theoretical frameworks such as environmental enrichment hypothesis, cardiovascular fitness hypothesis, and cognitive component skills theory shed light on the cognitive underpinnings of football. These insights carry significant implications for educational practices, emphasizing the potential of football as a holistic approach to cognitive development in children and adolescents. Integrating football into educational curricula and designing targeted interventions can leverage the specific cognitive demands of the sport, providing a promising avenue for optimizing cognitive outcomes in young individuals. Future research should test whether integrative drills that combine cognitive elements with basic skill practice are more effective than skill practice alone, providing a new direction for cognitive development interventions.

## Data Availability

The original contributions presented in the study are included in the article/supplementary material, further inquiries can be directed to the corresponding author.
